# Fate of Ingested Aristolactams from *Aristolochia chilensis* in *Battus polydamas archidamas* (Lepidoptera: Papilionidae)

**DOI:** 10.3390/insects4040533

**Published:** 2013-10-11

**Authors:** Alejandro Urzúa, Angel Olguín, Rocío Santander

**Affiliations:** Facultad de Química y Biología, Universidad de Santiago de Chile, Casilla 40, Correo 33, Santiago 9170022, Chile; E-Mail: rocio.santanderm@usach.cl

**Keywords:** *Aristolochia chilensis*, aristolactams, *Battus polydamas archidamas*, larvae, sequestration, excretion, metabolization

## Abstract

We performed a sequestration study of aristolactams (ALs) from *Aristolochia chilensis* in *Battus polydamas archidamas* (Lepidoptera: Papilionidae) by examining the AL content of the plant, fifth instar larvae, osmeterial secretion, pupae, exuviae and feces. Aristolactam-I (AL-I) and aristolactam-II (AL-II) present in *A*. *chilensis* are sequestered by fifth instar larvae of *B*. *polydamas archidamas*. There is a preferential sequestration of AL-II, or a more efficient metabolization and excretion of AL-I, by the larva. No ALs were found in the osmeterial secretion, pupae and exuviae; in addition, little AL-I and no AL-II were found in larval frass. The two lactams, particularly AL-I, are extensively metabolized to other products in the larva. A reasonable hypothesis is that the ingested ALs are oxidized to their respective aristolochic acids.

## 1. Introduction

The neotropical papilionid genus *Battus* is monophagous, feeding only on plants of the genus *Aristolochia* at the larval stage [1,2,3]. In central Chile, *Battus polydamas archidamas* Boisd. (Papilionidae, Troidini), the only representative species of the genus in Chile, uses *Aristolochia chilensis* Bridges ex Lindl. as its host plant [4].

Several aristolochic acids (AAs), 4,5-dioxoaporphines and aristolactam-I (AL-I) have been isolated from *A*. *chilensis* [5,6,7]. AAs are sequestered by larvae and have been found in wild adults and pupae of *B*. *polydamas archidamas* reared on *A*. *chilensis* [8,9].

Analysis of the AA content in pupae of *B*. *polydamas archidamas* has shown that, of the total ingested AA mixture, only 2% remains in the body tissues, 25% is excreted and the rest is metabolized to other unidentified compounds [9].

The plants in the family *Aristolochiaceae* also contain aristolactams (ALs), a group of aporphinoids with a phenanthrene backbone in their structure [10,11,12].

The similar structural relationship between ALs and AAs suggests that AAs are directly derived from ALs by oxidation. A biogenetic route has been proposed in which a 4-hydroxyaporphinic alkaloid is oxidized to a 4,5-dioxoaporphine, which, by CO extrusion, generates an AL and, after oxidation, produces aristolochic acid (AA) [13].

Although the sequestration of AAs by larvae of the genus *Battus* in the Troidini tribe (Papilionidae) has been studied [9,14,15,16,17], there is no information regarding the possible sequestration of ALs. These compounds are ingested by the larvae from their host plants, but nothing is known about their excretion and metabolism, or if they play any role as semiochemicals.

In the light of these observations, we studied the sequestration of ALs from *A*. *chilensis* by *B*. *polydamas archidamas* by examining the AL content of the plant, fifth instar larvae, osmeterial secretion, pupae, exuviae and frass to evaluate whether sequestration occurs, whether the sequestration varies during development from larvae to pupae and whether ALs are excreted in the frass and exuviae. We used high-performance liquid chromatography with diode-array detection (HPLC-DAD), which is widely used for the identification and quantification of ALs in herbal medicines [18,19,20,21,22], and gas chromatography-mass spectrometry (GC-MS).

## 2. Experimental Section

### 2.1. Insects and Plants

Fieldwork was performed at Cuesta Lo Prado (15 km west of Santiago, 33°28'S, 70°56'W, 750 m above sea level) from mid-December 2011 to mid-January 2012. Five plants of *A*. *chilensis* of similar size and phenological stage, and containing a single group of second instar larvae (14 ± 2 larvae), were marked. The larvae feeding on each of these plants were monitored every two days until they reached the fourth day of the fifth instar. Then, 50 larvae were collected and separated, and the aerial parts of each of the five plants (20 g, fresh weigh) (samples P1 to P5) were immediately immersed in warm methanol (100 mL) and subjected to extraction and analysis.

The larvae collected from each plant were divided into two groups: half of them (group I) were transferred to five potted plants in the laboratory and allowed to develop further until they pupated (two days); the other half (group II) were weighed (average fresh wt. per larva, 2.01 ± 0.3 g), and, after starvation for 24 h to avoid contamination by food remains in the gut, killed by freezing. In this manner, five groups of five larvae each (Samples L1 to L2) were obtained.

The groups of larvae that were allowed to develop further by feeding on potted plants were killed by freezing two days after they had changed to pupae. In this manner, five groups of five pupae each were obtained and subjected to extraction and analysis. The exuviae produced during the transformation from larvae to pupae were also submitted to extraction and analysis.

In addition, 10 s instar larvae collected in the field on the same group of studied plants, were transferred to potted plants in the laboratory and allowed to develop further had their frass collected and dried every day. Before they pupate the osmeterial secretion was extracted with small pieces of filter paper which were subjected to extraction with methanol and the extracts were directly analyzed. The total dried frass material (9.1 g) was subjected to extraction with methanol and analyzed.

### 2.2. Extraction and Isolation of Aristolactams

Each sample was ground in a blender with methanol (100 mL), and the resulting pulp was solvent-extracted at room temperature for 24 h. The suspensions were filtered and subjected to a second extraction under the same conditions, and the solvent was removed under reduced pressure. All methanolic extracts were processed in the same manner to isolate the fractions that contained aristolactams (ALs). The extracts were diluted with 20 mL of 3% NaHCO_3_, warmed to 40 °C, left standing for 6 h and then extracted with CHCl_3_ (4 × 20 mL). The basic solution was discarded, and the combined CHCl_3_ extracts were dried and concentrated under reduced pressure. The residue was subjected to preparative TLC on pre-coated plates of silica gel 60 F_254_ Merck (1.0 mm thickness, 20 × 20 cm) in CH_2_Cl_2_-MeOH (95:5). The mixtures of ALs were detected under UV irradiation at 364 nm as intense fluorescent bands (light blue) and were eluted from the plates using CHCl_3_-MeOH (80:20). The extracts obtained from each fluorescent band were analyzed by HPLC-DAD and GC-MS.

### 2.3. HPLC-DAD Analysis of Aristolactams

**Figure 1:**
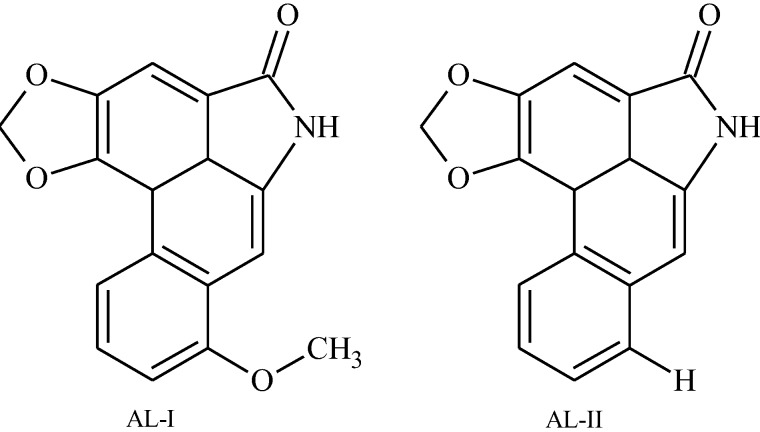
Aristolactam structures.

The Aristolactams, AL-I (1) and AL-II (2), detected in this study were characterized using reference compounds obtained from *Aristolochia argentina* [13] and by the reduction of a mixture of aristolochic I and II with Zn/AcOH (R751619, Sigma-Aldrich) [23]. The UV spectra and retention times of the ALs detected in the larvae, plant and frass. samples were coincident with the standards of AL-I (1) and AL-II (2), Figure 1. Quantification was based on chromatograms of the samples detected at 254 nm, by means of straight lines drawn from plots of peak areas against concentrations.

The aristolactam fractions were directly injected (20 µL) into an analytical HPLC (Waters 600), using a reverse-phase Symmetry column (5 μm particle size; 25 × 0.46 cm). Gradient elution was performed using a mobile phase consisting of 0.1% acetic acid in water (solution A) and 0.1% acetic acid in acetonitrile (solution B) as follows: 0–5 minute, isocratic elution with 70% A/30% B; 5–45 minute, linear gradient from 70% A/30% B to 55% A/45% B. Detection was made using a Waters 2996 diode-array-detector (DAD), and spectra were recorded at wavelengths between 200 and 800 nm.

### 2.4. GC-MS Analysis of Aristolactams

Qualitative analyses were conducted on a GC-MS Thermo Scientific (trace GC ultra, MS ISQ) apparatus with a DB-5 MS fused silica capillary column (60 m × 0.25 mm × 0.25 µm film thickness). The operating temperatures used were: injector 270 °C, detector 300 °C and column oven 100 °C up to 300 °C (5 °C minute^−1^). Helium at 1.3 mL minute^−1^ was used as carrier gas. 

## 3. Results and Discussion

**Figure 2:**
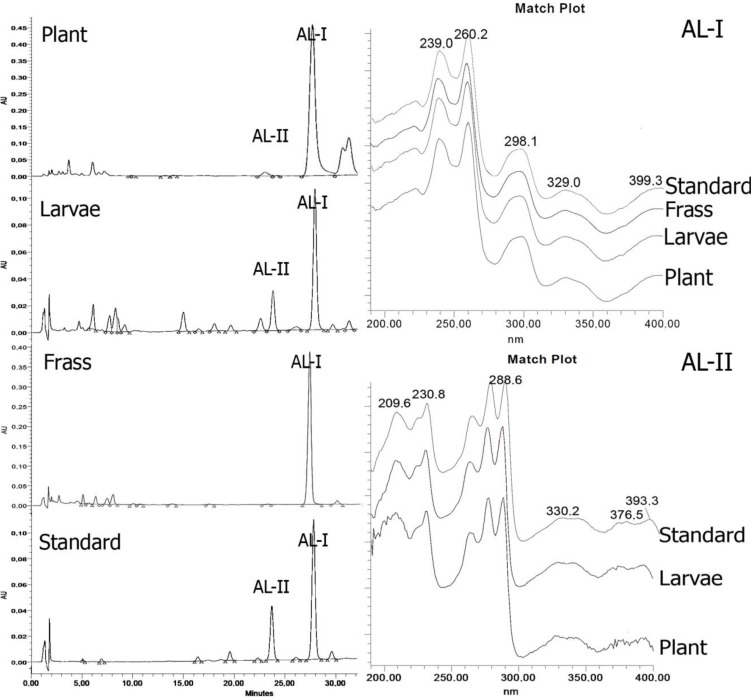
Representative high-performance liquid chromatography with diode-array detection (HPLC-DAD) of the aristolactam (AL) fractions from *A*. *chilensis* leaves and stems, fifth instar larvae of *B*. *polydamas archidamas*, frass and a standard mixture of AL-I and AL-II.

Representative HPLC analyses and UV spectra of the AL fractions obtained by preparative thin layer chromatography (PTLC) from *A*. *chilensis* leaves and stems, fifth instar larvae of *B*. *polydamas archidamas* and frass as well as a standard mixture of AL-I and AL-II are presented in Figure 2. HPLC analyses of the AL fraction from *A*. *chilensis* leaves and stems show that AL-I is the main lactam and AL-II is a minor component. Two other compounds (retention times of 29.201 and 29.736 minute), presumably aristolactams as suggested by their UV spectra, were detected in the plant extracts but were not identified. AL-I and AL-II were detected in the extracts from the fifth instar larvae of *B*. *polydamas archidamas* and in the frass sample. 

The identity of AL-I and AL-II was confirmed by GC-MS measurement of the AL in samples from *A*. *chilensis*, *B*. *polydamas archidamas* larvae, and frass. The mass spectra were coincident with those of the standards. All samples showed m/z [M^+^] 293.3 and m/z [M^+^] 263.4.The mass spectra of the aristolactams were identical to the spectra of the standards of AL-I and AL-II, and the fragmentation was also similar to that reported in the literature [13] (Table 1).

The possible excretion of ALs by the larvae was investigated by analyzing their frass, which were collected until they pupated. Excretion of AL-I, but not AL-II, was detected. Furthermore, no ALs were detected in the exuviae produced after the larvae pupated.

The amounts of AL-I and AL-II found in each of the analyzed samples of *A*. *chilensis* (samples P1-P5), in the fifth instar larvae of *B*. *polydamas archidamas* (samples L1-L5) and in the frass from 10 larvae are given in Table 2. 

No significant variation was found in the ability to sequester AL-I and AL-II in the fifth instar larvae samples L1-L5 from *B*. *polydamas archidamas*. Because these amounts correspond to extracts from five larvae, the average values per larva may be obtained by dividing the values by 5. These values are listed in Table 3. Previous laboratory studies [9], allow us to assume that one average *B*. *polydamas archidamas* larva (2.01 ± 0.3 g of fresh weight) consumes around 20 g of fresh plant during its life cycle, the total amount of AL-I and of AL-II that it ingests corresponds to the values given in Table 2 for P1-P5. Dividing the amount of lactam actually found in an average larva by the corresponding amount it ingested may then give the percentage of lactam retained by the larva before it pupated. These percentages are also listed in Table 3.

**Table 1:** Gas-chromatography mass spectrometry of aristolactam I and II.

Lactams	RT	KI	MS (m/z, %)
AL-II	34.0	3112.7	M^+^ 263 (95.5), 207.3 (38.9), 179.2 (35.8), 177.2 (43.1), 152.3 (75.3), 151.1 (38.5), 150.1 (100), 131.2 (40.0), 76.1 (28.9), 75.2 (55.4).
AL-I	41.3	3383.9	M^+^ 293 (95.6), 278.3 (96.7), 250.3 (54.0), 207.3 (38.6), 166.3 (69.2), 164.1 (100), 139.3 (59.8), 124.6 (40.1), 137.1 (55.7), 73.1 (48.1).

RT: retention time; KI: calculated Kovats indexes.

**Table 2:** Amount of aristolactams (in µg) in samples of the host plant *Aristolochia chilensis* and the fifth instar larvae of *Battus polydamas archidamas* and their frass.

Lactams	Aristolactams in host plant samples (P1-P5) ^a^						Frass
	P1	P2	P3	P4	P5	Mean ± SD	
**AL-I(1)**	1,207	1,184	1,140	1,270	1,185	1,197.2 ± 47.4	
**AL-II(2)**	26.8	27.9	30.1	26.5	27.4	27.74 ± 1.43	
**Aristolactams in larvae samples (L1-L5) ^b^**							
	**L1**	**L2**	**L3**	**L4**	**L5**	**Mean ± SD**	
**AL-I(1)**	291.5	289.5	278.5	285	290	286.9 ± 5.3	745.8 ^c^
**AL-II(2)**	70.5	76.5	75.5	78.1	74	74.92 ± 2.9	–

^a^ Amounts from 20 g of fresh plant; ^b^ total amount of ALs sequestered by samples of five larvae; ^c^ total amount of lactams excreted in the frass of 10 larvae.

**Table 3:** Amount of lactams retained by one average larva (average weight of 2.01 ± 0.3 g) from sample groups L1-L5 before pupating. Values between brackets correspond to the percentage of lactam retained by one larva relative to the total amount it ingested.

Lactams	Amount in µg					
	L1	L2	L3	L4	L5	Mean ± SD
**AL-I**	58.3 (4.8%)	57.9 (4.9%)	55.7 (4.9%)	57.0 (4.3%)	57.0 (4.5%)	57.38 ± 1.1
**AL-II**	14.1 (52.6%)	15.3 (54.8%)	16 (53.2%)	15.9 (58.9%)	14.8 (54.0%)	15.22 ± 0.8

The percentage of AL-I retained by an average larva varies from 4.3 to 4.9% of the total AL-I ingested from *A*. *chilensis* during development. By contrast, the percentage of AL-II retained by an average larva is much higher, varying from 55.7 to 58.3% of the total AL-II ingested from the plant during development.

### 3.1. Discussion

Future studies on the sequestration of ALs by larvae of the genus *Battus* should take into account the possibility that ALs may be formed from AAs in the larvae by a detoxification mechanism in a manner similar to that occurring in mammals [24,25]. This would require a distinction to be made between the ALs sequestered and the ALs synthesized by the larva. However, when a mixture of AA-I and AA-II was added to the artificial meridic diet of *B*. *polydamas archidamas*, AAs were partially transformed into AA-Ia and AA-IVa and there was no evidence for the presence of ALs, which is contrary to the hypothesis that aristolactams are created from AAs by larva [26]. We might assume that all the aristolactams found in the larvae, or larval frass originated from the ALs ingested from the plant. 

No ALs were found in the osmeterial secretion, this might indicates that the lactams are accumulated only in the body of the larvae.

The results listed in Table 3 indicate that the amount of AL-I retained by the larvae is consistently larger, by a factor of nearly four, than the amount of AL-II. This partly reflects the AL distribution in the plant because AL-I is much more abundant (by a factor of nearly 40) than AL-II. In reality, a comparison of the AL-I/AL-II ratio in the plant and in the larvae suggests a significant reduction of this ratio (almost ten-fold) after uptake by *B*. *polydamas archidamas*, which may reflect either a preferential sequestration of AL-II or a more efficient metabolization and excretion of AL-I by the larva. Little AL-I (74.6 µg out of 1,197.2 ± 47.41 µg ingested from the plant) and no AL-II were found in the larval frass. The percentages in Table 3 indicate that these two lactams, particularly AL-I, are extensively metabolized to other products by the larva.

**Figure 3:**
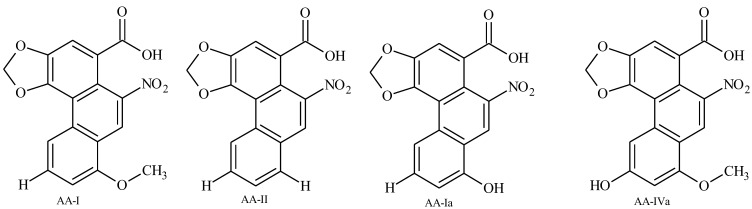
Aristolochic acid structures.

No ALs were found in the pupae and exuviae. In this stage of development, the only way to eliminate ALs is in the exuviae, so the only explanation for these results is that the ALs are also metabolized during the change from larvae to pupae. A possible hypothesis is that ingested AL-I is oxidized to AA-I. This hypothesis is plausible because it would explain the presence of AAs in Troidini swallowtails that accumulate aristolochic acids in their tissues despite their feeding from *Aristolochia* species that do not contain AAs. This explanation seems more reasonable than the suggestion by some authors that these AAs could be synthesized *de novo* from a 1-benzyltetrahydroisoquinoline precursor by the Papilionidae. This is very unlikely because the enzymes responsible for isoquinoline alkaloid biosynthesis are highly specific and restricted to plant families containing these compounds [27]; on the contrary, oxidation processes of xenobiotics have been documented in *Battus* in the oxidation of AAs into phenolic analogues [26]. In addition, because AA-I is the most toxic and anti-feedant AA [28,29,30], the preferential biotransformation of AL-I to AA-I, compared with AL-II, may reveal a unique mechanism of defense by the insect. The data in Table 3 suggest that *B*. *polydamas archidamas* larvae might extensively oxidize the ingested aristolactams I and II to the corresponding acids; and metabolizes these compounds in a selective way, with a preferential conversion of AL-I into the most toxic AA-I as a means of protection against predators (Figure 3).

## 4. Conclusions

AL-I and AL-II present in *A*. *chilensis* are sequestered by fifth instar larvae of *B*. *polydamas archidamas*. There was a preferential sequestration of AL-II, or a more efficient metabolization and excretion of AL-I, by the larva. No ALs were found in the pupae and exuviae; in addition, little AL-I and no AL-II were found in larval frass. The two lactams, particularly AL-I, are extensively metabolized to other products in the larva. A reasonable hypothesis is that the ingested ALs are oxidized to their respective AAs. This hypothesis might explain the presence of AAs in Papilionidae that accumulate aristolochic acids in their tissues, despite their feeding from *Aristolochia* species that do not contain AAs.

## References

[1] Feeny P., Price P.W., Lewinsohn T.M., Fernandez J.W., Benson W.W. (1991). Chemical constraints on the evolution of swallowtail butterflies. Plant-Animal Interactions: Evolutionary Ecology in Tropical and Temperate Regions.

[2] Weintraub J.D., Scriber J.M., Tsubaki Y., Lederhouse R.C. (1995). Host plant association patterns and phylogeny in the tribe troidini. Swallowtail Butterflies: Their Ecology and Evolutionary Biology.

[3] Silva-Brandão K.L., Freitas A.V.L., Brower A.V.Z., Solferini V.N. (2005). Phylogenetic relationships of the new world troidini swallowtails (lepidoptera: papilionidae) based on COI, COII, and EF-1α genes. Mol. Phylogenet. Evol..

[4] Peña L., Ugarte A. (1997). Las Mariposas de Chile.

[5] Urzúa A., Freyer A.J., Shamma M. (1987). Aristolodione, a 4,5-Dioxoaporphine from *Aristolochia chilensis*. J. Nat. Prod..

[6] Urzúa A., Rojas V. (1990). Constituents of *Aristolochia chilensis*. Fitoterapia.

[7] Urzúa A., Santander R., Sotes G. (2009). Aristolochic acids from *Aristolochia bridgesii*, a host-plant of *Battus polydamas archidamas*. J. Chil. Chem. Soc..

[8] Urzúa A., Salgado G., Cassels B.K., Eckhardt G. (1983). Aristolochic acids in *Aristolochia chilensis* and the aristolochia-feeder *Battus archidamas* (lepidoptera). Coll. Czech. Chem. Comm..

[9] Urzúa A., Rodríguez R., Cassels B.K. (1987). Fate of ingested aristolochic acids in *Battus archidamas*. Biochem. Syst. Ecol..

[10] Kumar V., Poonam, Prasad A.K., Parmar V.S. (2003). Naturally occurring aristolactams, aristolochic acids and dioxoaporphines and their biological activities. Nat. Prod. Rep..

[11] Bentley K.W. (2006). β-Phenylethylamines and the isoquinoline alkaloids. Nat. Prod. Rep..

[12] Kuo P.-C., Li Y.-C., Wu T.-S. (2011). Chemical constituents and pharmacology of the *Aristolochia species*. J. Tradit. Comp. Med..

[13] Priestap H.A. (1985). Seven aristololactams from *Aristolochia argentina*. Phytochemistry.

[14] Urzúa A., Priestap H.A. (1985). Aristolochic acids from *Battus polydamas*. Biochem. Syst. Ecol..

[15] Fordyce J.A. (2000). A model without a mimic: Aristolochic acids from the California pipevine swallowtail, *Battus philenor hirsuta*, and its host plant, *Aristolochia californica*. J. Chem. Ecol..

[16] Klitzke C.F., Brown K.S. (2000). The occurrence of aristolochic acids in neotropical troidine swallowtails (lepidoptera: papilionidae). Chemoecology.

[17] Priestap H.A., Velandia A., Johnson J., Barbieri M. (2012). Secondary metabolite uptake by the *Aristolochia*-feeding papilionoid butterfly *Battus polydamas*. Biochem. Syst. Ecol..

[18] Jou J.-H., Li C.-Y., Schelonka E.P., Lin C.-H., Wu T.-S. (2004). Analysis of the analogues of aristolochic acid and aristolactam in the plant of *Aristolochia* genus by HPLC. J. Food Drug Anal..

[19] Zhang C., Wang X., Shang M., Yu J., Xu Y., Li Z., Lei L., Li X., Cai S., Namba T. (2006). Simultaneous determination of five aristolochic acids and two aristololactams in *Aristolochia* plants by high-performance liquid chromatography. Biomed. Chromatogr..

[20] Yuan J., Nie L., Zeng D., Luo X., Tang F., Ding L., Liu Q., Guo M., Yao S. (2007). Simultaneous determination of nine aristolochic acid analogues in medicinal plants and preparations by high-performance liquid chromatography. Talanta.

[21] Yuan J., Liu Q., Zhub W., Ding L., Tang F., Yao S. (2008). Simultaneous analysis of six aristolochic acids and five aristolactams in herbal plants and their preparations by high-performance liquid chromatography-diode array detection-fluorescence detection. J. Chromatogr. A.

[22] Chen H.J., Li X., Chen J.W., Guo S., Cai B.C. (2010). Simultaneous determination of eleven bioactive compounds in *Saururus chinensis* from different harvesting seasons by HPLC-DAD. J. Pharm. Biomed. Anal..

[23] Priestap H.A., de los Santos C., Quirke J.M. (2011). Identification of a reduction product of aristolochic acid: implications for the metabolic activation of carcinogenic aristolochic acid. J. Nat. Prod..

[24] Schmeiser H.H., Bieler C.A., Wiessler M., van Ypersele de Strihou C., Cosyns J.-P. (1996). Detection of DNA adducts formed by aristolochic acid in renal tissue from patients with Chinese herbs nephropathy. Cancer Res..

[25] Zhou S., Koh H.-L., Gao Y., Gong Z.-Y., Lee E.J.D. (2004). Herbal bioactivation: The good, the bad and the ugly. Life Sci..

[26] Pinto C.F., Urzúa A., Niemeyer H.M. (2011). Sequestration of aristolochic acids from meridic diets by larvae of *Battus polydamas archidamas* (papilionidae: troidini). Eur. J. Entomol..

[27] Sato F., Hashimoto T., Hachiya A., Tamura K., Choi K-B., Morishige T., Fujimoto H., Yamada Y. (2001). Metabolic engineering of plant alkaloid biosynthesis. Proc. Natl. Acad. Sci. USA.

[28] Alali F.Q., Tawaha K., Shehadeh M.B., Telfah S. (2006). Phytochemical and biological investigation of *Aristolochia maurorum* L.. Z. Naturforsch C.

[29] Lajide L., Escoubas P., Mizutani J. (1993). Antifeedant activity of metabolites of *Aristolochia albida* against the tobacco cutworm, *Spodoptera litura*. J. Agric. Food Chem..

[30] Jeude S.E. (2011). Quality *vs.* quantity: The effect of aristolochic acids on preference and performance of a non-specialist herbivore. Purs. J. Undergrad. Res. Univ. Tenn..

